# Relationships between muscle mass, strength and regional bone mineral density in young men

**DOI:** 10.1371/journal.pone.0213681

**Published:** 2019-03-08

**Authors:** Thibault Sutter, Hechmi Toumi, Antoine Valery, Rawad El Hage, Antonio Pinti, Eric Lespessailles

**Affiliations:** 1 EA 4708—I3MTO Laboratory, University of Orleans, Orleans, France; 2 Department of Rheumatology, Regional Hospital of Orleans, Orleans, France; 3 Department of Medical Information, Regional Hospital of Orleans, Orleans, France; 4 Department of Physical Education, University of Balamand, EL-Koura, Lebanon; Ehime University Graduate School of Medicine, JAPAN

## Abstract

**Purpose:**

Although the relationship between body composition and bone mineral density (BMD) is well established, the relative contribution of appendicular lean mass (ALM) and fat mass (FM) to BMD has been rarely evaluated in young men.

**Methods:**

We assessed 100 young men (age: 24.4±2.8 years, BMI: 23.4±2.81 kg/m^2^). Appendicular lean mass index (ALM/H^2^) (ALMI), fat mass index (FM/ H^2^) (FMI), percentage of body fat, BMD at lumbar spine (LS), total hip (TH), femoral neck (FN) and whole body (WB) were measured using DXA. Muscle strength was evaluated by handgrip strength. Pearson’s correlations and interactions between all variables were assessed using stepwise regression analyses.

**Results:**

ALM index (ALMI) was positively correlated with BMD at all sites (r = 0.62 for WB p<0.05, r = 0.54 for FN p<0.05, r = 0.64 for TH p<0.05, r = 0.56 for LS p<0.05) whereas FMI was not correlated to BMD values. Stepwise regression analyses showed that ALMI produced a significant and positive influence on BMD (β = 0.07 for WB p<0.001, β = 0.04 for FN p<0.001, β = 0.06 for TH p<0.001). Conversely, FMI was negatively associated with BMD at all sites (β = -0.02 for WB p<0.001, β = - 0.03 for FN p<0.001, β = - 0.03 for TH p<0.001, β = - 0.07 for LS p<0.001). Handgrip strength and BMDs were significantly and positively associated at all sites.

**Conclusions:**

Our data suggest that BMD was positively associated with ALMI while negatively with FMI. We confirm that ALMI is the strongest factor associated with BMD in a population of young men.

## Introduction

Illustrating muscle-bone [1,2] and fat-bone interactions [3] changes in body composition occur throughout life. These changes have important metabolic and functional consequences as illustrated in cachexia and sarcopenia [4,5]. In addition, changes in body composition including lean mass anf fat mass lead to bone changes in aging population that can lead to sarcopenic obesity and osteoporosis through multifactorial and complicated relationships [6–8].

Although the relationship between Bone Mineral Density (BMD) and body composition has been widely described in young women, post-menopausal women and in elder women fewer data are available on young men [9–11].

The findings of these three previous studies reported a significant moderately positive correlation between lean body mass with whole body BMD (WBBMD) but only one study assessed the correlations between specific regional bone sites and body composition parameters in thirty six young men [11]. Furthermore, none of these studies investigated the potential role of the volume of physical activity in these relationships. Indeed, several factors are known to have an effect on the BMD including physical training, physical exercise but also physical activity [12]. Concomitant obesity also affects bone and muscle outcomes [13]. After a peak and a plateau phase both muscle mass and strength along with bone mass suffer from a gradual degradation due to the senescence process [14]. Although in young adults, muscle mass can be built fairly rapidly, in older individuals this ability is compromised [15]. In addition, both bone and muscle tissues are known to share common factors such as metabolic, genetic and hormonal factors including sexual steroids, insulin, IGF-I and GH which play a role in bone fragility and sarcopenia [16]. There is also controversy concerning the link between fat mass and BMD, since some studies found a negative association [17] while others reported positive correlations [9,10].

Therefore, the aim of this study was to investigate the relationships between body composition (fat mass, lean mass) and BMD both at the whole body and at specific bone sites (lumbar spine and the hip) in a group of young men.

## Materials and methods

### Subjects

The present study included 100 ambulatory young French Caucasian men aged 20–30 years. We choose this age range based on the study by Baxter-Jones et al. indicating that total body BMC reached a plateau 7 years post peak height velocity representing about 20.5 years of age in boys [18]. We did not nclude mixed race or transgender subjects.

These young men were recruited between March and May 2016 in Orleans and the surrounding suburbs through advertising in regional press releases as well as transmission of this press release among health professionals (physiotherapists, pharmacies …) and through regional businesses. An information email (which included the items listed in the press release) was relayed both to the occupational medicine department at Orleans hospital and to the students of the University of Orleans. General exclusion criteria were non-Caucasian origin and the presence of external or internal artifacts that could affect the region being scanned and that compromise the analysis or interpretation of the bone density scan at the hip, spine or total body.

The subjects received an allowance of 40 euros for their participation in the study.

The study was approved by the local Ethical Committee of Tours (No. 2015-A01786-43) on March 02, 2016 and written informed consent was obtained from all subjects.

### Anthropometric measurements

Anthropometric measurements were carried out in our research laboratory and performed by the same operator (TS). Body weight was assessed using a digital scale (SECA 709, Hamburg, Germany) to the nearest 0.1kg with participants dressed in light clothes. In order to limit variability, body height was measured with a Holtain stadiometer to the nearest 0.1cm (Holtain Ltd., Crymych, UK). Body mass index (BMI) was calculated by body weight / body height^2^ (kg/m^2^). Left calf circumference (CC) was measured with the participant supine; his left knee raised and flexed 90° to the thigh. The measurement was carried out a second time after repositioning. The circumference of the hip was assessed using a tape measure at the widest portion of the buttocks. Waist circumference was measured at the midpoint between the lower margin of the last palpable rib and the top of the iliac crest.

Tobacco use, health history, use of medications known to affect bone health and usual occupation of the participant were also collected.

### Grip strength measurement

Handgrip strength was measured by a Jamar hydraulic hand dynamometer (Jamar Plus +, Sammons Preston, Bolingbrook, Il, USA). This dynamometer was calibrated as previously described [19], and used according to the recommandations of the American Society of Hand Therapists in the standardized condition. The measurement position adopted was the seated position with shoulders adducted and neutrally rotated, elbow flexed at 90°, the forearm in the neutral position and the wrist between 0 and 30° of dorsiflexion. Participants were encouraged to squeeze as hard and as tightly as possible during 3 to 5 seconds for each of the measurements. Two sets of measurements were carried out. Each set consisted of six measures. The force test was carried out as follows: three measurements on the right hand and three measurements on the left hand with 15 seconds of recovery between each measurement. The first series of measurements was carried out before the densitometric analyses and the second series of measurements after these analyses. We retained the best value of these two series. The maximum value was recorded as recommended previously [20] based on the fact that the maximum value is less likely to be affected by the number of trials than the mean.

### Physical performance tests

All participants underwent a series of tests using the Short Physical Performance Battery (SPPB) [21]. Briefly, this included balance, gait speed and the repeated chair stand tests. The latter is a timed test requiring participants to rise consecutively for five times from a chair without using their arms and return to the seated position. For each event, a performance score (ranging from 0 to 4 points for the 3 phases of the test) is given and the addition of the scores of all the items produces an overall performance. A score below 8 is in favor of sarcopenia [21]. Physical activity was also assessed using the short form IPAQ questionnaire [22]. Each score from these three exercises can be extracted individually. For our statistical analyzes, we chose to extract gait speed in order to study the impact of this value on bone mineral density. The short form IPAQ questionnaire is a self‐reported questionnaire aiming at evaluate the participation in physical activity during a seven‐day period. Physical activity is determined by nine items that assess the time spent in vigorous and moderate activity, walking and sedentary activity [22]. Subjective estimates of exercise intensity were determined using activity codes and the metabolic equivalents of task (MET) for all physical activities performed over the last seven days [23]. The physical activity score for weekly energy expenditure was expressed in MET-minutes per week.

### Whole body composition assessment

A dual-energy X-ray absorptiometry (DXA) scanner (Hologic Discovery A, Hologic Inc., Bedford, MA, USA) was used to measure BMD (g/cm^2^) at the whole body (WB), lumbar spine (LS), total hip (TH) and femoral neck (FN). The coefficient of variation for our device during measurement on a standard phantom was less than 1% for FN, WB and LS BMD. We further assessed total body fat mass (FM, kg), total lean mass (LM, kg) and the percentage of fat mass according to manufacturer-recommended procedures. Analysis of scans was performed using software (Hologic Discover, version 13.4.2) and the regions of interest for all sites were placed manually by trained study staff according to a standard analysis protocol. All analyses were checked by one researcher for consistency (TS). Appendicular lean mass (ALM, kg) was determined by the sum of arms and legs. The appendicular lean mass index (ALMI) was obtained from the appendicular lean mass (ALM)/height^2^ (kg/ m^2^) according to the official position of ISCD (International Society for Clinical Densitometry) recommendations [24]. The fat mass index (FMI) was calculated by the fat mass (FM)/height^2^ (kg/m^2^) [24]. The percentage of body fat mass was calculated as fat mass divided by total mass x 100. Visceral adipose tissue (VAT in g) was also obtained by the adipose indices measurement provided by the same software. Subjects wore underwear during densitometric measurements. The quality control program included both a daily spine phantom scan and at least once per week body composition phantom scan as recommended by the manufacturer’s protocol.

### Statistical analyses

Means and standard deviations (SD) were calculated for anthropometric measures. The normality of the variables was assessed by the Kolmogorov-Smirnov’s test. Correlation analyses were performed with Pearson’s r or with Spearman’s rho when variables were not normally distributed, as was the case for instance with fat mass [25]. In order to determine the best explanatory variables a correlation matrix was constructed to avoid redundancy and minimize collinearity bias estimated with the variance inflation factor [26]. We constructed an explanatory model based on the results of univariate analyzes (p < 0.05). We also introduced in our models variables including p <0.2 (physical activity, age, height and other anthropometric parameters than CC) to study their impacts in the models. The models were consolidated and optimized using a stepwise method (Akaike’s information criterion [27]). The normal distribution of residues was verified. The results of the multiple linear regression analyses were expressed as β coefficients and standard deviation error. The coefficient of determination (R^2^ adjusted) was also calculated. Associations were considered significant at p value < 0.05. The 95% confidence intervals were also calculated for each factor. Statistical analyses were performed using the R software Core Team (2015, R Foundation for Statistical Computing, Vienna, Austria, Version 0.99.896, RStudio, INC).

## Results

### Descriptive characteristics of the population

One hundred men with a mean age of 24.4±2.8 years and mean BMI of 23.4±2.8 kg/cm^2^ were included in the analysis. None of the participants had a chronic disease or medication known to affect bone. Detailed participant characteristics are shown in Table 1.

**Table 1:** Characteristics of the study population (n = 100).

Variables	mean±SD
Age (year)	24.4±2.81
Height (cm)	176.4±6.19
Weight (kg)	72.9±9.34
Body mass index (kg/m^2^)	23.4±2.81
Calf circumference (cm)	36.8±0.13
Waist circumference (cm)	82.4±7.66
Hip circumference (cm)	94.9±7.03
Whole body BMD (g/cm^2^)	1.23±0.10
Femoral neck BMD (g/cm^2^)	1.01±0.14
Total hip BMD (g/cm^2^)	1.11±0.14
Lumbar spine BMD (g/cm^2^)	1.07±0.13
Total lean mass (kg)	61.91±7.48
Appendicular lean mass (kg)	28.09±3.83
Appendicular lean mass index (kg/m^2^)	9.04±1.22
Total fat mass (kg)	11.40±4.67
% Fat mass	15.28±4.87
Fat mass index (kg/m^2^)	3.65±1.48
Visceral adipose tissue (g)	273±80.49
Handgrip strength (kg)	49.32±7.90
Short physical performance battery test	11.95±0.26
Metabolic Equivalent of Task (MET-min/week)	6892±2047
Gait speed (m/s)	3.74±0.67

The percentage of overweight participants (BMI ≥ 25kg/m^2^) was 28%. Among these participants 22% were students, 77% worked in various professions (medical doctors, mechanics, engineers, technicians) and 1% was unemployed. The Orleans population census undertaken in 2014 showed that the population of young men in this region was distributed as follows: 71%, 26% and 3% were respectively workers, students and unemployed people. There were 11% of smokers in our population. We found significantly lower values of BMD at all sites in smokers versus non-smokers (data not shown).

The mean results of handgrip strength measurements were 48.83 ± 8.17 kg for the first set of measurements and 49.81 ± 8.32 kg for the second set. The best value of the two sets corresponded to the dominant hand of the participants. The values of MET were in the following range [1890–9990] (MET-min /week) and the mean was 6891±2047 (MET -min /week).

### Correlation between BMD and other body composition parameters

Table 2 shows the correlations between BMD at various sites and body composition parameters, anthropometric measurements, handgrip strength, MET and gait speed.

**Table 2:** Pearson's correlation coefficient (CI 95%) of BMD sites with selected body composition, strength and physical performance parameters.

Variable (units)	Whole body BMD	Femoral neck BMD	Total hip BMD	Lumbar spine BMD
Age (years)	0.09(-0.102; 0.286)	-0.10 (-0.294;0.094)	-0.14 (-0.327;0.057)	0.14 (-0.051;0.333)
Poids (kg)	0.36 (0.177;0.520)*	0.30 (0.110;0.469)*	0.36 (0.177;0.521)*	0.35 (0.175;0.519)*
BMI (kg/m^2^)	0.40 (0.232;0.561)*	0.32 (0.141;0.493)*	0.40 (0.227;0.557)*	0.42 (0.249;0.573)*
Waist circumference (cm)	0.12 (-0.071;0.315)	0.08 (-0.112;0.277)	0.08 (-0.114;0.275)	0.18 (-0.016;0.364)
Hip circumference	0.16 (-0.033;0.349)	0.04 (-0.153;0.238)	0.09 (-0.105;0.283)	0.19 (0.003;0.380)
Calf circumference (cm)	0.34 (0.159;0.507)*	0.38 (0.200;0.537)*	0.42 (0.250;0.574)*	0.29 (0.135;0.488)*
ALMI (kg/m^2^)	0.62 (0.514;0.747)*	0.54 (0.399;0.675)*	0.64 (0.538;0.761)*	0.56 (0.466;0.717)*
FMI (kg/m^2^)^a^	-0.15 (-0.336;0.047)	-0.12 (-0.316;0.069)	-0.14 (-0.336;0.047)	-0.12 (-0.294;0.094)
MET (MET-Min/week)	0.18 (-0.008;0.370)	0.16 (-0.036;0.346)	0.22 (0.026;0.400)	0.21 (0.016;0.392)
Handgrip strength (kg)	0.35 (0.166;0.512)*	0.28 (0.180;0.421)*	0.36 (0.177;0.521)*	0.29 (0.100;0.460)*
Score SPPB	0.19 (-0.0018; 0.376)	0.16(-0.035; 0.347)	0.14 (-0.051;0.332)	0.13 (-0.065;0.320)
Gait speed (m/s)	-0.12 (-0.317;0.068)	-0.02 (-0.215;0.176)	-0.03 (-0.230;0.161)	-0.11 (-0.300;0.088)

BMI : body mass index

ALMI : appendicular lean mass index

FMI : fat mass index

*p<0.05

a : Results of spearman correlation

Correlation analyses showed that ALMI was moderately and positively correlated with BMD at all the bone sites. The strongest correlation was found between ALMI and TH (r = 0.64; p<0.05).

Conversely, no correlation was found between FMI and BMD parameters. BMI was weakly and positively associated with BMD at all sites. CC and handgrip strength were weakly correlated significantly with BMD parameters whatever the bone site considered (Table 2). The highest correlation between CC, grip strength and BMD was observed at the total hip site (r = 0.42, p<0.05 and r = 0.36, p<0.05 respectively). We extrapolated the value of the gait speed of the SPPB test to study the impact of this variable, which seems relevant to us. Gait speed was not significantly associated with BMD. Globally we did not find any significant relationships between the volume of physical activity and body composition parameters. We observed, however, a weak correlation between ALMI and the MET (r = 0.33; p<0.05). We found significant correlations between CC and BMD at all sites explaining. Significant correlations were found between CC and ALMI (r = 0.61; p<0.05) and between CC and handgrip strength (r = 0.34; p<0.05).

### Regression analyses of body composition, muscle strength and BMD

Multivariable linear regression models demonstrated an association between BMD and independent factors (Table 3).

**Table 3:** Multiple linear regression analyses between handgrip strength, age, height, FMI, ALMI, calf circumference and BMDs (Model with the stepwise method Akaike’s information criterion [27]).

Dependent variables				
Independent variables	Whole Body BMD	Femoral neck BMD	Total Hip BMD	Lumbar spine BMD
Handgrip strength (kg)	-0.0019 (0.001)	-	-	-0.0030(0.0017)
Age (year)	0.0058 (0.002)	-	-0.0062 (0.003)	0.0074*(0.0038)
Height (cm)	0.0039**(0.001)	-	-	0.0038*(0.002)
FMI (kg/m^2^)	-0.0234***(0.005)	-0.0330***(0.009)	-0.0324***(0.008 )	-0.0753***(0.009)
ALMI (kg/m^2^)	0.0721***(0.008)	0.0476***(0.012)	0.0636***(0.010)	-
Calf circumference (cm)	-	0.0166*(0.006)	0.0150**(0.005)	-
Residual standard error	0.076	0.114	0.100	0.102
R^2^ adjusted	0.50	0.36	0.52	0.44

Results expressed as β coefficients and standard deviation error

ALMI : appendicular lean mass index

FMI : fat mass index

***P<0.001

**P<0.01

*P<0.05

Comparing the models with the Akaike’s information criterion allowed us to select the robust models for each explanatory variable. The most robust models were selected for each bone site studied based on the standard error and the lowest total error and highest R^2^ adjusted. In our stepwise multivariable model including handgrip strength, FMI, ALMI and CC, we observed that ALMI remained significantly associated with BMD parameters at WB, FN and TH (β = 0.072, β **=** 0.047 and β = 0.063) respectively, all p values <0.001. Although CC was significantly associated with BMD at FN and TH (β = 0.016 and β = 0.015) respectively, CC was not a contributing factor to explain BMD at the WB and LS sites. FMI was associated with BMD at all sites but its contribution was lower than that of ALMI and was consistently negatively linked to BMD. VAT was not included in our regression analyses. Handgrip strength was not included in models of contribution to hip BMD. Although not the dominant independent contributor, handgrip strength non significantly contribute to multivariate spine and whole body BMD regression models (Table 3). Overall, the variables included in these models accounted for 36 to 54% of the variability in BMD. A combination of CC, FMI and ALMI with respective increasing contributions in the model gave the best R^2^ value at the total hip bone site.

## Discussion

Our study confirmed that among the body composition variables and baseline anthropometric characteristic, ALMI was consistently a significant and positive independent contributor to whole body and hip BMD in healthy young men aged 20 to 30 years. Lean body mass has been found to be positively correlated with BMD in women of different age groups [28–31]. In men, our findings are consistent with most previous studies on the relationships between body composition and BMD showing in multivariate linear regression models that lean mass is uniquely associated with the largest proportion of variance of WBBMD [6,10,32,33]. Blain et al. showed [32] that ALMI was the most significantly correlated factor (r = 0.39, p <0.0001) and associated (β = 0.00283, p <0.0001) to the FN BMD. Verschuren et al. found that ALMI was also strongly associated with all bone sites (β = 0.317, p <0.001 for WBBMD, β = 0.3730, p <0.001 for FN, β = 0.433, p <0.001 for TH, β = 0.294, p <0.001 for LS) [33]. However, in older populations the appendicular skeletal muscle mass factor explains between 15% to 20% of the variability at the femoral neck [32,33]. The positive association between ALMI and BMD might reflect the direct mechanical effects (muscle contractions and resulting movements) of muscle tissue on bone through their regulation by both genetic and life style factors such as physical activity [12].

In contrast, FMI was not correlated with BMD in univariate analyses. However, despite the lack of a significant relationship between FMI and BMD parameters in univariate analyses, FMI contributed negatively as an independent factor in multivariate models at all sites. Our study is in agreement with Taes and al. [17] where a negative contribution of fat mass at all bone sites was observed in healthy young men. However, the relationship between FM and BMD is controversial, as most of the studies have found no association or an inverse relationship [17,34–37] while some studies have shown a positive relationship [10]. Interestingly, in the latter study, although the authors found a positive role for fat mass in peak bone mass attainment in young male adults, it accounted for only 1.8% of the variation in whole body BMD. Gender differences have been consistently reported in the relationship between fat mass and bone mass [10,38]. This difference may be explained by gender-specific effects of sex hormones on muscle and bone. Furthermore, this complex relationship between bone parameters and muscle may differ with the age and pubertal growth stage of the population studied but also with the anatomical sites measured. Low grade inflammation, associated with increased inflammatory cytokines in the case of excess adipose tissue, produces a negative impact on bone metabolism [39]. In addition the skeletal muscle metabolism might be altered in obese subjects due to the increase in lipotoxicity and insulin resistance [40]. Thus, in the present study, we specifically assessed visceral adipose tissue by DXA and looked for its association with bone parameters but we did not find any correlation either in univariate or multivariate analyses (data not shown). Conversely, visceral fat measured by computerized tomography in 100 healthy young women had a negative effect on the femoral bone [41]. Due to the weak age range of the subjects, we did not find any association between ALMI and this variable.

Our study also highlighted that handgrip strength was significantly but moderately correlated with BMD at all sites. Handgrip strength contribution in our multivariate model analysis was also limited to the variation of WB BMD and LS BMD. In a young male adult population (n = 36) aiming at describe the influence of muscle strength and lean body mass on BMD, the magnitude of the correlation between muscle strength and BMD was slightly lower than between lean mass of regional and whole body composition to BMD [11]. In a young European male population (21 years old), neither handgrip strength nor isokinetic knee flexion or extension strength variables were associated with total body BMD [6]. Conversely, in a population of middle-aged and elderly European men (40–79 years), handgrip strength was significantly associated with whole body and total hip BMD [33]. In a much older population (men were 57.3 ± 10.2 years old), significant correlations between knee extensor strength and bone mineral densities were found in men at both lumbar spine and femoral neck (Pearson’s correlation coefficients were 0.08 and 0.16, respectively) [42].

Theoretically, physical activity might underlie the relationship between muscle mass and strength indices and BMD. In our study, physical activity was not captured in our statistical model to explain BMD parameters. Again, this lack of relationship may be due to the low range of variation in the volume of physical activity engaged in by our subjects as assessed through the short form IPAQ questionnaire. It is also possible that the short IPAQ questionnaire was not fully adapted to our population. In addition, our assessment of the amount of activity physical did not distinguish resistance exercise and endurance type exercise done by the participants.

Besides ALMI and handgrip strength, physical performance is also used for the diagnosis of sarcopenia [43]. We thus assessed gait speed, SPPB and Metabolic Equivalence of Task. However none of these parameters were significantly associated with BMD parameters in univariate or multivariate analyses. Although gait speed and SPPB are relevant parameters in the elderly, these two parameters were not useful in this young population in which every subject was able to walk quickly and to perform the SPPB tests completely.

Among different anthropometric parameters generally used in the evaluation of sarcopenia, the CC has been shown to be the most highly correlated with muscle mass [44]. In the present study, CC was a weak but significant parameter included in the multivariate models explaining TH and FN BMD. Calf circumference is an anthropometric variable which is correlated with appendicular skeletal muscle mass [44,45]. In addition it has been shown that leg physical function and particularly the sural triceps had a crucial role in the maintenance of the autonomy [46]. Consequently, it can be hypothesize that CC might have greater influence on BMD measurements located at the lower limb than at the lumbar spine or whole body.

Our study has strengths and limitations. The strengths are the assessment of volunteers young men from a semi-urban, semi-rural area in France with validated tools to measure both muscle and bone mass and handgrip strength. In accordance with the NHANES methodology the only exclusions in this cohort were made for reasons of DXA scan accuracy [47]. To the best of our knowledge, our study is the only one to have assessed the correlation between WBBMD but also peripheral sites of interest (FN, TH, LS) and body composition parameters in a population of young men. Nevertheless, we performed a cross-sectional study in a rather small population (n = 100), which precludes any conclusions about causal relationships. The tool for measuring physical performance (SPPB) was not adapted to our population. Furthermore we did not assess the history of physical activity in our population which might have been more relevant but prone to recall bias. Another limitation was the lack of information on nutrient supply particularly protein and vitamin D which play a significant role in mediating muscle metabolic function [48]. We did not collect information’s about nutrition or history of an eating disorder and history of limb fractures. Furthermore we did not measure bone-marrow fat which may also play a role in the relationship between bone, muscle and fat [49].

In conclusion, in this specific young men population, who attains peak bone mass, we confirmed that ALMI was an important contributor to BMD both at the whole body but also at conventional regional bone sites (TH, FN and LS).Other determinants not captured by muscle strength, muscle mass or physical performance might intervene in the bone phenotype. As muscle and bone are connected tissues with both mechanical and biochemical cross-talk [1,50] the weak but negative impact of FM on bone phenotype that we also found illustrates the possible role of adipokines in the complex relationship between bone, muscle and fat tissues. Research in this field should lead to better knowledge in order to reduce the continuing gap between fundamental data and clinical practice and recommendations concerning sports and physical activity.
